# Experimental investigation of novel ternary amine-based deep eutectic solvents for CO_2_ capture

**DOI:** 10.1371/journal.pone.0286960

**Published:** 2023-06-23

**Authors:** Hossam K. Abdrabou, Inas AlNashef, Mohammad Abu Zahra, Salim Mokraoui, Emad Ali, Mohamed K. Hadj-Kali

**Affiliations:** 1 Department of Chemical Engineering, Khalifa University, Abu Dhabi, United Arab Emirates; 2 Research and Innovation Center on CO_2_ and H_2_ (RICH), Khalifa University, Abu Dhabi, United Arab Emirates; 3 Chemical Engineering Department, College of Engineering, King Saud University, Riyadh, Saudi Arabia; Universiti Teknologi Petronas: Universiti Teknologi PETRONAS, MALAYSIA

## Abstract

This study investigates the effect of using water as a low-viscosity component in ternary amine-based deep eutectic solvents (DESs) on the physicochemical properties, thermal stability, and CO_2_ absorption capacity of the resulting DESs. It should be emphasized that water is a component of the ternary DES. The effect of water content in the DES, type of hydrogen bond acceptors (HBAs), hydrogen bond donors (HBDs), and HBA:HBD ratio on the above parameters was investigated. Moreover, the effect of temperature and pressure on the CO_2_ absorption capacity of DESs was predicted using the predictive model COSMO-RS. This model was also used to predict the CO_2_ solubility in the DESs and the results were compared with the experimental values. The results showed that the addition of small amounts of water, e.g., 5 and 10 wt% during preparation, can significantly decrease the viscosity of the resulting DESs, up to 25% at room temperature, while maintaining the high CO_2_ absorption capacity and high thermal stability. The ternary DESs based on MEA exhibited a high CO_2_ absorption capacity of 0.155–0.170 g CO_2_ / g DES. The ternary DESs were found to be thermally stable with a decomposition temperature of 125°C, which promotes the use of such solvents in post-combustion capture processes. Finally, COSMO-RS proved to be a suitable tool for qualitative prediction of CO_2_ solubility in DESs and demonstration of trends related to the effects of temperature, pressure, molar ratio, water content, HBD and HBA on CO_2_ solubility.

## Introduction

The increase in global energy consumption due to economic growth and urbanization relies heavily on the use of fossil fuels such as oil, coal, and natural gas, releasing enormous amounts of carbon dioxide (CO_2_) into the atmosphere. Assuming that greenhouse gas emissions continue to increase at the current rate, global warming is projected to reach 1.5°C between 2030 and 2052 [1]. Given the status of CO_2_ as a pollutant and greenhouse gas, intergovernmental regulations impose penalties on countries and factories to reduce and control their CO_2_ emissions. One mature and promising technology to reduce anthropogenic CO_2_ emissions is post-combustion carbon capture technology [2]. This technology is more often based on chemical or physical absorption processes. However, the predominant technique for CO_2_ capture is chemical absorption by aqueous solutions of amine-based solvents, such as monoethanolamine (MEA), diethanolamine (DEA), methyldiethanolamine (MDEA), piperazine (PZ), and 2-amino-2-methyl-1-propanol (AMP) [3].

Several research groups have studied the absorption of CO_2_ in these solvents (MEA, DEA and MDEA) to determine the absorption capacity, regeneration energy and reaction kinetics [4]. In general, a good solvent must have high CO_2_ absorption capacity, fast reaction kinetics, low regeneration energy, and negligible environmental impact. However, Zhao et al. [5] found that the degradation of MEA solution accounts for nearly 10% of the total cost of CO_2_ capture. Moreover, MEA requires high regeneration energy. In contrast, MDEA has a low regeneration energy, however, it has much lower reaction kinetics. To overcome these disadvantages, there have been several attempts to use amine mixtures to find a compromise between the different performance parameters. Indeed, Zhang et al. [6] developed a kinetic model of CO_2_ absorption in aqueous solutions of MDEA mixed with DEA based on a homogeneous activation mechanism. Glasscock et al. investigated CO_2_ absorption and desorption in aqueous solutions containing MDEA/MEA and MDEA/DEA. They concluded that the combined mass transfer/equilibrium model based on the zwitterion mechanism can successfully represent the CO_2_ mass transfer rate in the temperature range of the experiments [7]. Also, in a study conducted by Bruder et al. [8], it was found that a mixture of AMP and PZ had a higher loading capacity than AMP alone; however, undesirable precipitation can occur when high concentrations of PZ are added. In general, processes using aqueous amines have some limitations, such as high energy demand, corrosiveness, and degradation of some amines, which leads to foaming, fouling, performance degradation, and production of harmful gasses, resulting in environmental problems [9]. Therefore, in recent years, many research groups have investigated the use of new green solvents to replace aqueous amines in carbon capture processes. Examples of these potentially green solvents include ionic liquids (ILs) and deep eutectic solvents (DESs).

ILs have several interesting properties, including their wide liquid range, non-flammability, non-volatility, and thermal stability, which promote their use as greener solvents in many chemical and industrial processes [10], especially for CO_2_ absorption. Many research groups have investigated the use of different types of ILs for CO_2_ capture, e.g., Raeissi et al. [11] and Kumełan et al. [12]. It was found that some ILs have excellent absorption capacity for CO_2_ with low regeneration energy. Nevertheless, ILs have critical drawbacks that hinder their industrial application, including their high cost, toxicity in some cases, and the complex synthesis process that requires purification steps to remove impurities that could affect the properties of IL.

A new class of green solvents, called deep eutectic solvents (DESs), has shown promise for various applications [13]. These solvents share many similarities with ILs, e.g. low volatility, etc. However, based on the proper selection of their components, DESs have advantages over ILs, such as low cost, nontoxicity, ease of preparation, and biodegradability. In addition, DESs can be used for a variety of applications, including CO_2_ capture, due to their ability to be tuned by changing their constituents and the ratio of HBA:HBD [14, 15].

Several researchers have investigated the possibility of using DESs for absorbing CO_2_. For example, Lu et al. [16] reported the CO_2_ solubility in levulinic acid (LV) and furfuryl alcohol (FA) based DESs with different molar ratios and at different temperatures, where the HBA was fixed as choline chloride (ChCl). In general, it was found that ChCl: LV DESs exhibited better CO_2_ absorption capacity than ChCl: FA DESs. It was also found that as the molar ratio of LV or FA increased, the CO_2_ solubility increased. As expected, CO_2_ solubility increased with the increase of pressure and decrease of temperature. Li et al. [17] investigated CO_2_ solubility in choline chloride with urea DES (ChCl:urea) at 3 different molar ratios, namely 1:1.5, 1:2, and 1:2.5 at temperatures ranging from 313.15 K to 333.15 K. The results showed that ChCl:urea (1:2) had the highest CO_2_ solubility, while the other two molar ratios showed similar results. Moreover, the same trend reported by Lu et al. [16] regarding the effect of pressure and temperature on CO_2_ solubility was reported by Li et al. [17]. Sze et al. [18] studied the solubility of CO_2_ in ternary DESs. The prepared DESs consisted of choline chloride as HBA, glycerol as HBD, and 3 superbases, namely 1,5-diazabicyclo[4.3.0]-non-5-ene (DBN), 7-methyl-1,5,7-triazabicyclo[4.4.0]dec-5-ene (MTBD), and 1,8-diazabicyclo[5.4.0]undec7-ene (DBU). It was found that ChCl:Gly:DBN with 1:2:6 molar ratio had the best overall performance with a CO_2_ absorption capacity of 103 mg CO_2_/g DES. However, this system has a very high viscosity, ranging from 5450 to 34,613 mPa.s for the neat DES at 298.15 K. In a recent publication, Adeyemi et al. [19] investigated the solubility of CO_2_ in 3 different amine-based DESs at molar ratios of 1:6, 1:8 and 1:10. In all DESs, the HBA was choline chloride, while MEA, DEA and MDEA were used as HBDs. The solvent screening setup (SSS) and total organic carbon (TOC) analyzer were used to measure the CO_2_ absorption capacity in all DESs. The results showed that ChCl:MEA at a molar ratio of 1:10 had the highest CO_2_ absorption capacity among all the DESs tested. In all cases, as the molar ratio of amines increased, CO_2_ solubility increased. The results were compared with those obtained by using conventional DESs such as ChCl:glycerol. It was found that the amine-based DESs have much higher CO_2_ absorption capacity. Moreover, the performance of the amine-based DESs was compared with that of 30% wt aqueous amines. For ChCl:MEA DESs, the CO_2_ solubility was higher than that achieved by the respective 30 wt% amine aqueous solutions. However, CO_2_ solubility in MDEA 30 wt% aqueous solution was higher than that of ChCl: MDEA DES for all 3 molar ratios.

Wu et al. [20] used 1-ethyl-3-methylimidazolium chloride ([Emim]Cl) + imidazole DESs to selectively remove H_2_S from gas mixtures containing CO_2_ for applications in the natural gas industry. The authors compared the results with other liquid solvents and reported that [Emim]Cl + imidazole DES showed extremely high efficiency in H_2_S absorption and great selectivity over CO_2_.

Sarmad et al. [21] prepared five new three-component DES by functionalization of choline chloride-ethanolamine (1:7) DES using different types of amines: DEA, MDEA, piperazine and 1-(2-aminoethyl)piperazine. The solubility of CO_2_ in the studied DESs was measured at pressures up to 2 MPa and 298.15 K. The Redlich-Kwong equation of state was used to correlate the experimental data and determine the Henry’s law constant. The authors applied an analytical method to prove that all studied DESs exhibited chemical absorption of CO_2_. Haghbakhsh et al. [22] reported the use of a new alcoholic DES (choline chloride: Butane-1,2-diol (molar ratio 1:4)) for CO_2_ absorption in a temperature range from 303.2 to 333.2 K and at pressures up to 3.4 MPa. The authors reported the values of Henry’s constants at very dilute CO_2_ concentrations, in addition to the calculated values of standard enthalpies, entropies, and Gibbs free energies of dissolution.

Wang et al. [23] reported the use of amino acid DESs modified by the addition of triethanolamine (TEA) to the HBD to mask the protons and protect the amino groups. The authors investigated the effects of HBD modification of L-Arg DESs and L-Lys DESs on CO_2_ binding. The experimental data showed that CO_2_ loading reached 1.194 mol/mol when L-Arg:glycerol: TEA was in the ratio of 1:3:3, and 1.378 mol/mol when L-Lys: glycerol: TEA was in the ratio of 1:3:3, and both showed good regenerative ability. Al-Bodour et al. [24] studied the solubility of CO_2_ in four natural hydrophobic DESs based on carvone, cineole, thymol, and menthol at different temperatures and pressures. The results showed that the solubility reached that in aqueous amine solution at a pressure of about 30 bar. The physical absorption behavior was confirmed by analytical methods. This has a significant effect on the regeneration energy. Nagulapati et al. [25] used experimental solubility data from the literature for CO_2_, CO, CH4, H_2_, and N_2_ in ChCl/Urea DES to develop machine learning models for predicting the solubilities of various gases in ChCl/Urea DES. The hybrid model developed by the authors exhibited excellent prediction accuracy with low root mean square error values. Moreover, the predicted solubility data were regressed in Aspen Plus V11 to design the process for the separation of H_2_ from a gaseous feed mixture. Qian et al. [26] reported the use of DESs prepared with glycerol (Gly) and proline (Pro) as HBAs and MEA and MDEA as HBDs. It was found that the DESs based on MEA had better CO_2_ absorption capacity than the DESs based on MDEA. The authors used ethylene glycol to form ternary DESs. The authors found that the absorption kinetics and regeneration capacity were improved by the addition of EG. Gly: MEA:EG showed better CO_2_ capture performance than Pro: MEA:EG, reaching up to 0.25 g-CO_2_/g- DES at room temperature. In addition, Gly: MEA:EG showed excellent tolerance to water content and CO_2_ concentration, and there was no significant solvent loss after 10 cycles. The analytical methods showed that CO_2_ formed carbamate by reacting with the amine group in MEA and amino acids. In addition, CO_2_ was found to react with the–OH group in EG to form CO_3_^2-^/HCO_3_^-^. Pasha et al. [27] investigated the CO_2_ absorption performance of five novel diamine functionalized DESs in microstructured reactors with metal foams as fillers. It was found that the N-methyl-1,3-propanediamine (MAPA) functionalized DES showed remarkable absorption performance without significant increase in viscosity. The CO_2_ capacity and CO_2_ absorption of this DES reached 0.78 mol CO_2_/mol diamine and 98% at gas-liquid flow ratios of 640 and 240, respectively. Moreover, the results showed that the MAPA-functionalized DES exhibited low heat of absorption and remarkable regeneration ability. The authors reported that the overall rate constant and absorption flux of this DES were higher than most amine-functionalized DESs used previously. They concluded that the use of this DES in microreactors has great potential for process intensification in CO_2_ absorption. In a recent study, Cheng et al. [28] reported the mechanism of CO_2_ absorption by DESs based on ethylene glycol and protic IL, namely protonated MEA imidazolium ([MEAH][Im]). Analytical techniques showed that the interactions between CO_2_ and DESs [MEAH][Im]- EG (1:3) and that CO_2_ reacted not only with the amine group of MEA, but also with the deprotonated EG to form carbamate and carbonate, respectively. The authors proposed two mechanisms for the reaction between CO_2_ and DESs. It was concluded that the absorption mechanism found in this work was different from that of other DESs formed by protic ILs and EG.

Another important parameter that negatively affects the performance and use of a solvent in many applications is its viscosity. Since DESs share several common properties with ILs, including high viscosity, it is imperative to reduce the viscosity of DESs to reduce operating costs in the industry and improve mass transfer. Most of the above DESs used for CO_2_ capture have high viscosity, including the amine-based binary DESs studied by Adeyemi et al. [19]. Therefore, the aim of this work is to find novel amine-based ternary DESs with viscosity significantly lower than that of amine-based binary DESs. The proposed amine-based ternary DESs use low viscosity solvents such as water, which acts as the ternary component.

## Materials and methods

### Materials and sample preparation

The ternary DESs were prepared by a procedure similar to that described by Abbott et al. [13]. The source and purity of the chemicals used are summarized in Table 1.

**Table 1:** Source and purity of the chemicals used in this work.

Chemical	CAS number	Purity (*wt*%)	Source
Choline Chloride	67-48-1	≥ 99.0	Sigma- Aldrich
Ethanolamine	141-43-5	≥ 98.0	Sigma- Aldrich
Diethanolamine	111-42-2	≥ 98.0	Sigma- Aldrich
Methyl Diethanolamine	105-59-9	≥ 99.0	Sigma- Aldrich
Tetrabutylammonium Bromide	1643-19-2	≥ 98.0	Sigma- Aldrich
Tetrabutylphosphonium Bromide	3115-68-2	≥ 98.0	Sigma- Aldrich

Based on the molar ratio of HBA:HBD and the required weight percentage of water, the correct amounts of all 3 components were added to a properly sealed vial. The vial was then heated to about 353 K and stirred for several hours until the mixture became homogeneous and clear. Table 2 shows the main DESs prepared in this work.

**Table 2:** List of DESs prepared in this work.

	DES	Molar Ratio + Solvent Content
1	ChCl-MEA + Water	1:6 + (2.5 wt% water)
2	ChCl-MEA + Water	1:6 + (5 wt% water)
3	ChCl-MEA + Water	1:6 + (7.5 wt% water)
4	ChCl-MEA + Water	1:6 + (10 wt% water)
5	ChCl-MEA + Water	1:6 + (12.5 wt% water)
6	ChCl-MEA + Water	1:8 + (5 wt% water)
7	ChCl-MEA + Water	1:10 + (5 wt% water)
8	ChCl-DEA + Water	1:6 + (5 wt% water)
9	ChCl-MDEA + Water	1:6 + (5 wt% water)
10	TBAB-MEA + Water	1:6 + (5 wt% water)
11	TBPB-MEA + Water	1:6 + (5 wt% water)

### Characterization

The melting points of the DESs investigated in this work were measured by differential scanning calorimetry (DSC 131 evo, SETARAM, Switzerland) with a temperature accuracy of +/- 0.1°C and a precision of +/- 0.05°C. Water content was measured using a Karl Fischer titrator (GRS Scientific/Aquamax KF Coulometric). The viscosity and density of all prepared DESs were measured as a function of temperature using Anton Paar SVM 3001 viscosity and density meter. Viscosities were measured in the range of 0.2 mPa.s to 30,000 mPa.s with a repeatability of 0.1% and densities in the range of 0.6 to 3 g/cm^3^ with a repeatability of 5x10^-3^ g/cm^3^. Thermal stability of the samples was determined using the Perkin Elmer Thermogravimetric Analyzer (TGA 4000) with a temperature accuracy of +/- 1°C and a balance accuracy of +/- 0.02%.

### CO_2_ absorption capacity

**Fig 1:**
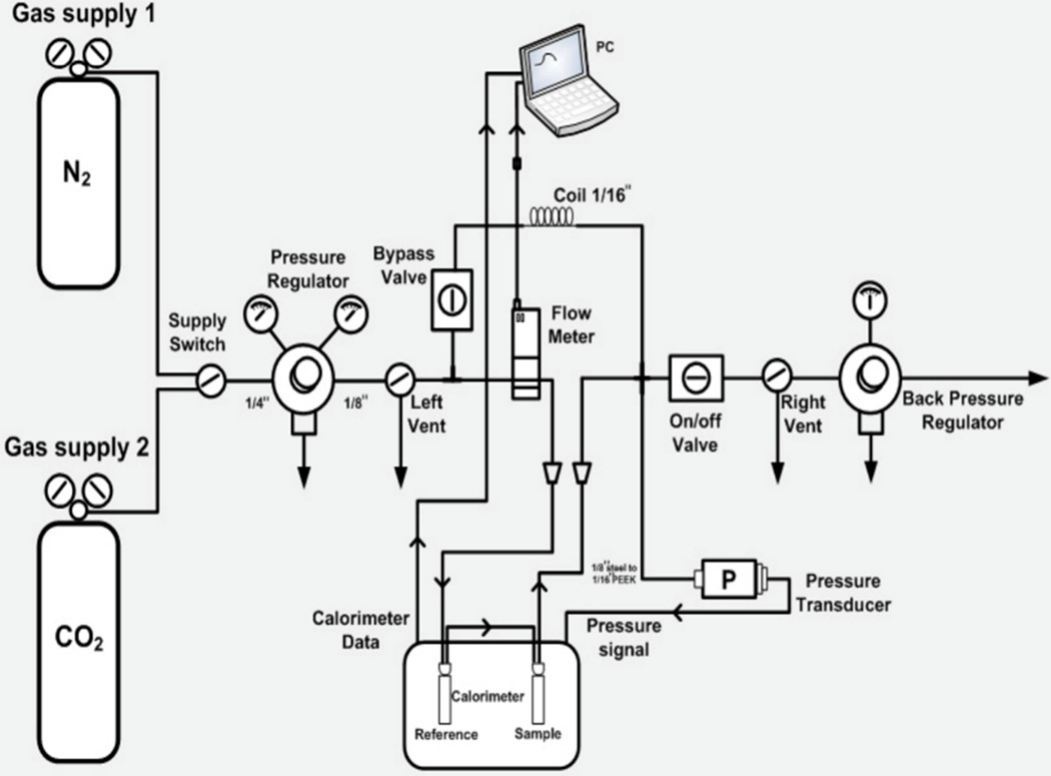
Schematic diagram of the micro-reaction calorimeter [21].

The CO_2_ absorption capacity and heat of absorption of the DESs were measured using the Thermal Hazard Technology micro-reaction calorimeter (μRC). The schematic diagram of the experimental setup is shown in Fig 1. The setup consists of a nitrogen gas cylinder and a CO_2_ gas cylinder. The gas supply can be easily switched between the two cylinders via the supply switch. The gas flow is controlled by a flow meter connected to the calorimeter and the μRC control and analysis software on the computer. A pressure transducer connected to a pressure regulator is used to measure and display the pressure of the gas during the process.

For each experiment, 0.5 g of the sample is injected into the vial using a syringe. The vial is properly sealed with its lid and then placed in the calorimeter with the appropriate tweezers. The μRC control and analysis software was then turned on and the process parameters were entered. The process temperature was set to 40°C and the pressure to 1 atm. The experiment was then started and a plot of power versus time was displayed in the software. More details about the experimental protocol are appended at the end of this manuscript.

### COSMO-RS calculation methodology

The Conductor like Screening Model for Real Solvents (COSMO-RS) was used to determine the effects of temperature and pressure changes on the CO_2_ absorption of DESs [29–32]. Two steps were required in order to proceed with the COSMO-RS simulation. First, all involved components were created on the TURBOMOLE program using TmoleX software and a geometry optimization of each component was performed at the DFT energy level with the def-TZVP basis set. At the end of this first step, a single point calculation was conducted and the.*cosmo* files were generated. In the Second step, the COSMOthermX was used for the prediction of the CO_2_ solubility of the DESs using the parameterization file BP_TZVP_19.ctd [33].

Furthermore, there are 3 different ways of representing DESs in COSMOtherm: (i) the electroneutral method, (ii) the ion pair method and (iii) the meta file method. In this work, the electroneutral approach was adopted in which each DES’ component is treated as a distinct individual species. This means that when the molar ratio of the components of the DES is considered, the cation and the anion of the salt would be considered as individual components. For example, if the molar ratio of ChCl:Urea is 1:2, that would be entered in COSMOthermX as 1:1:2 for chlorine cation, chloride anion and urea, respectively.

The original expressions for the chemical potential and activity coefficient were derived by Klamt and Eckert [30], and they were also reported elsewhere [34].

## Results and discussion

### Melting point

**Fig 2:**
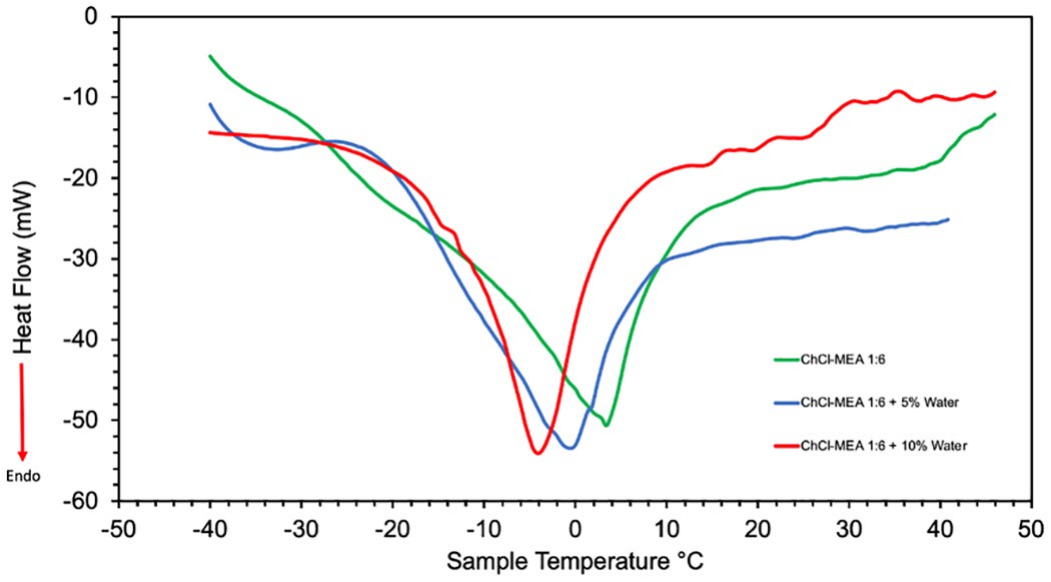
DSC Thermogram of neat ChCl-MEA (1:6) DES and with 5% and 10% water content.

The DSC thermograms of ChCl-MEA (1:6) and ChCl-MEA (1:6)-water DESs are shown in Fig 2. The melting point of each sample was determined from the peak of the respective melting curve. The results were first validated by measuring the melting point of ChCl- MEA (1:6) DES and comparing it with the value reported by Mjalli et al. [35]. There was an excellent agreement between the two values, about 4°C in this study while that reported by Mjalli et al. was 3.84°C. Subsequently, the melting points of ChCl- MEA (1:6) with 5% water and 10% water were determined to be -0.5°C and -4°C, respectively.

The melting points of the 3 DESs are below room temperature, which is desirable for the applications of such solvents. It is also observed that the melting point decreases with increasing water content in DES. It is well known that the formation of hydrogen bonds between the components of DES, in addition to charge delocalization, leads to a significant decrease in the melting point of the mixture compared to that of the individual components [13]. Thus, as the water content in the prepared ternary DESs increases, more hydrogen bonds are formed and further charge delocalization occurs and subsequently lowering the melting point.

### Viscosity and density

**Fig 3:**
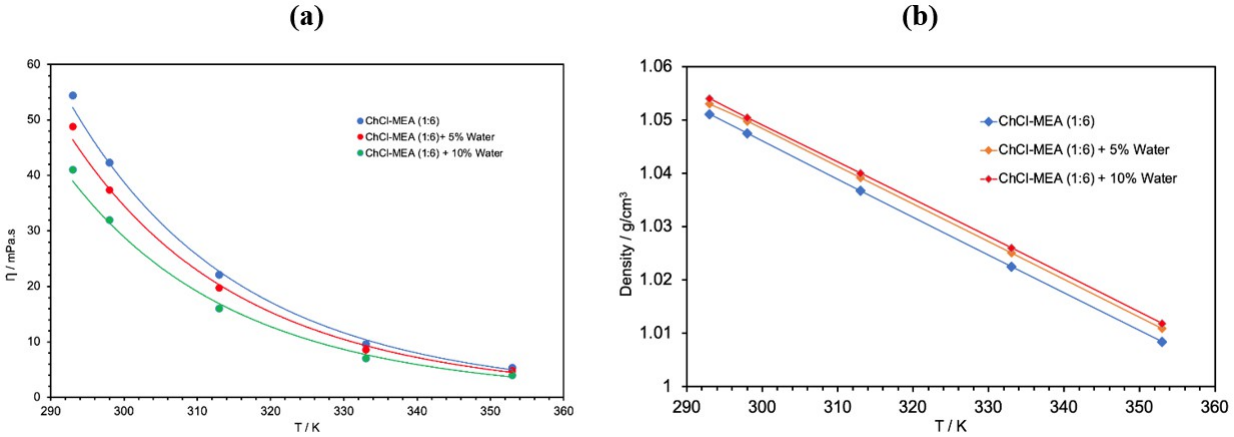
Effect of water content on the viscosity and density of the ChCl-MEA (1:6) DESs.

The viscosities and densities of the prepared DESs were measured as a function of temperature, from 293 K to 353 K, using the Anton Paar SVM 3001. The obtained experimental measurements are provided as supporting Information (S1 and S2 Tables). Fig 3 shows the effects of changing the water content in the ternary DESs on the viscosity and density. It should be noted that the viscosity of the solvent for CO_2_ absorption is very important not only to overcome mass transfer limitations and increase the solubility of CO_2_ but also because the viscosity of the solvent increases dramatically with the increase of the solubility of CO_2_ to the degree that it might cause technical problems in industrial application [36]. By lowering the viscosity of the DES, the viscosity of the CO_2_ saturated DES will be much lower than that for CO_2_ in the binary amine DES.

As expected, the viscosity and density of all DESs decrease as the temperature increases. It is also observed that the viscosity of the ternary DESs decreases with increasing water content. This shows that the effect of the presence of a low viscosity component such as water will overweigh the effect of the increase in the hydrogen bonding and eventually will lead to the decrease in the viscosity of the DESs compared to the binary DESs. This is in agreement with that reported in the literature by other groups [37]. The decrease in viscosity by introducing water as a component in the DES can be seen in other types of amine-based DESs. Table 3 illustrates the decrease in viscosity at room temperature when adding 5% water to each binary amine-based DES.

**Table 3:** Viscosity of different amine based DESs at room temperature.

	ChCl-MEA (1:6)	ChCl-MEA (1:6) + 5% Water	ChCl-DEA (1:6)	ChCl-DEA (1:6) + 5% Water	ChCl-MDEA (1:6)	ChCl-MDEA (1:6) + 5% Water
**Viscosity (mPa.s)**	51.71	48.8	567	434.14	139.8	123.16
**Reference**	Adeyemi et al. [36]	This work	Adeyemi et al. [36]	This work	Adeyemi et al. [36]	This work

In fact, it can be noticed that the decrease in viscosity is about 5.6%, 23.4% and 12%, respectively, for MEA, DEA and MDEA based DESs. It is worth noting that the percentage decrease of the viscosity of the ternary DES is larger for high viscosity binary DES. On the other hand, it is observed that the density increases with the increase of water content. However, the increase is much higher at 5% than at 10% for the same DES. The complex hydrogen bonds formed between the water and the other constituents of the DES lead to a contraction of the volume and consequently to an increase in density [38].

**Fig 4:**
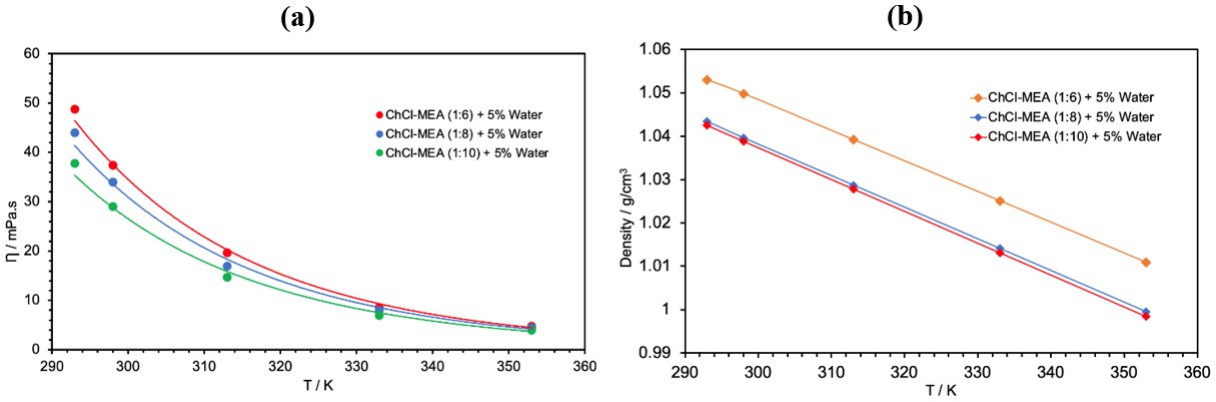
Effect of (HBA:HBD) molar ratio on viscosity and density of ChCl-MEA- 5% water DESs.

The effect of changing the HBD:HBA molar ratio on the viscosity and density of the ternary DESs was also investigated. For this purpose, 3 different molar ratios (1:6, 1:8 and 1:10) were tested for ChCl- MEA- water (5%) DESs. Fig 4 shows that as the molar ratio of MEA in the DES increases, the viscosity and density decrease. The same behavior was reported for the binary DES ChCl- MEA. For example, at 298 K, the viscosity and density decreased by about 22.5% and 2%, respectively, when the molar ratio increased from 1:6 to 1:10. This could be due to the lower viscosity and density of MEA compared to ChCl. Therefore, it is expected that the viscosity and density of the resulting DES will decrease when more MEA is added. As shown in Fig 4, the viscosity and density of all 3 DESs showed inverse proportionality with temperature.

**Fig 5:**
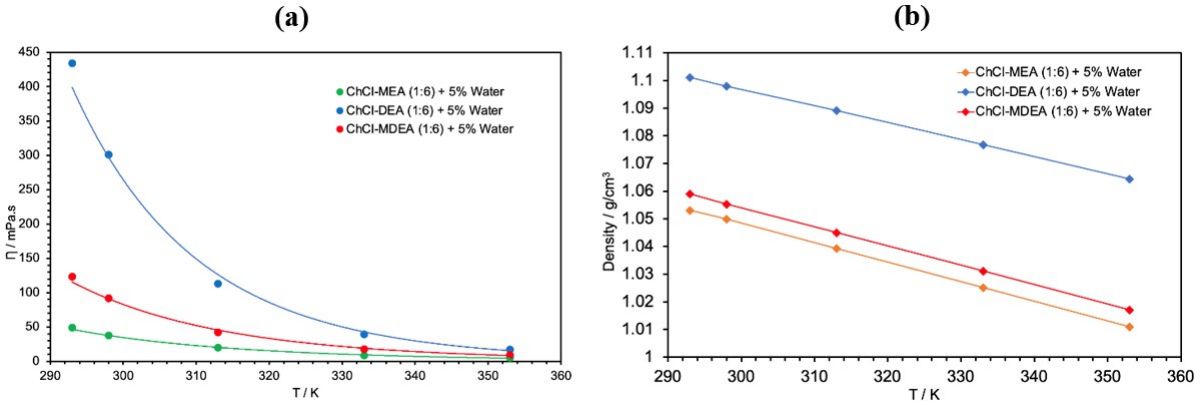
Effect of HBD type on the viscosity and density of ChCl-based DESs + 5% water.

Fig 5 shows the effect of the type of amine (MEA, DEA or MDEA) on the viscosity of the DESs as a function of temperature. DEA based DES has the highest viscosity and density over the whole temperature range with the values 301.2 mPa.s and 1.0979 g/cm^3^ at 298 K, while MEA based DES has the lowest viscosity and density with the values 37.4 mPa.s and 1.0388 g/cm^3^ at the same temperature. This may be attributed to the high viscosity and density of DEA compared to MEA and MDEA. Another explanation could be related to the hole theory [39]. MDEA and DEA based DESs have higher viscosity and density than MEA due to their larger molecules. This also means that they have a larger ionic radius, resulting in a smaller free volume and thus higher viscosity and density due to the limited mobility of the molecules [40]. These results support the use of MEA as HBD over DEA and MDEA in the CCS applications.

The viscosity and density experimental values of all prepared DESs at different temperatures can be found in the supplementary materials.

### CO_2_ absorption

**Fig 6:**
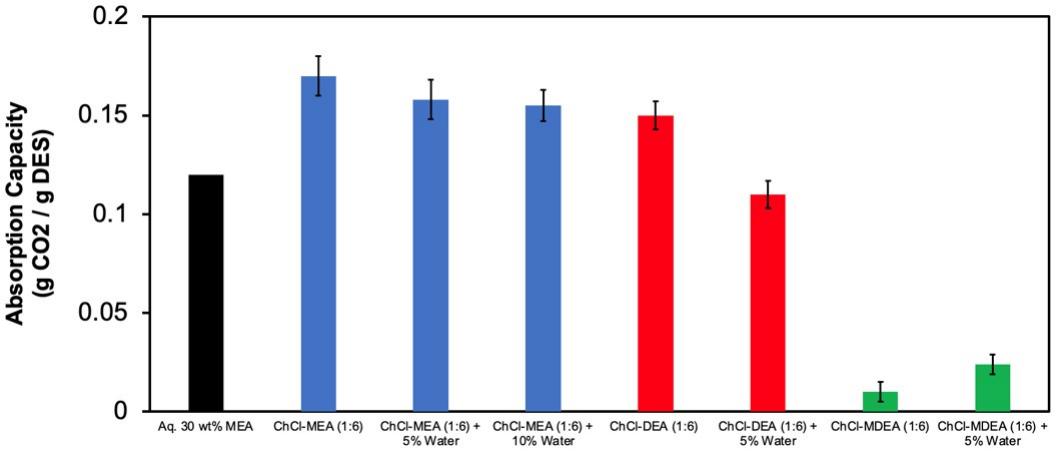
Effect of water content on CO_2_ absorption compared to 30 wt% aqueous MEA.

**Fig 7:**
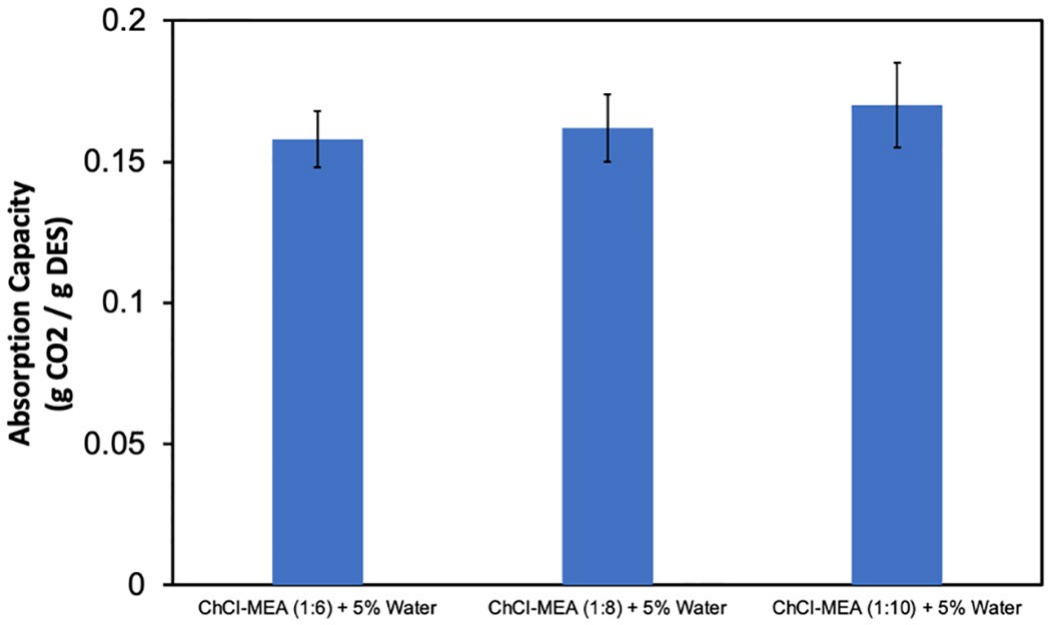
Effect of (HBA:HBD) molar ratio on CO_2_ absorption capacity of the ChCl-MEA-5% water DESs.

**Fig 8:**
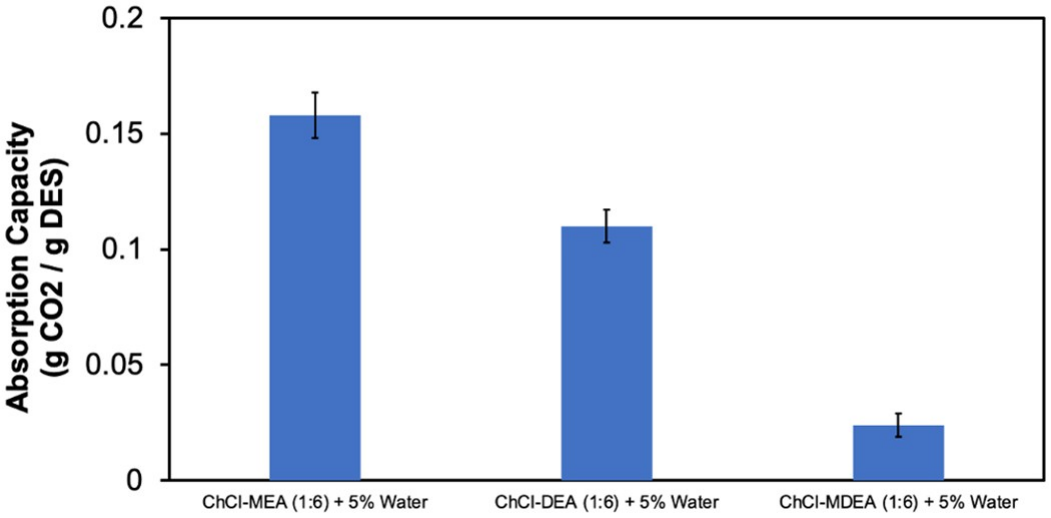
Effect of HBD type on CO_2_ absorption capacity of the ChCl-based DESs + 5% water.

**Fig 9:**
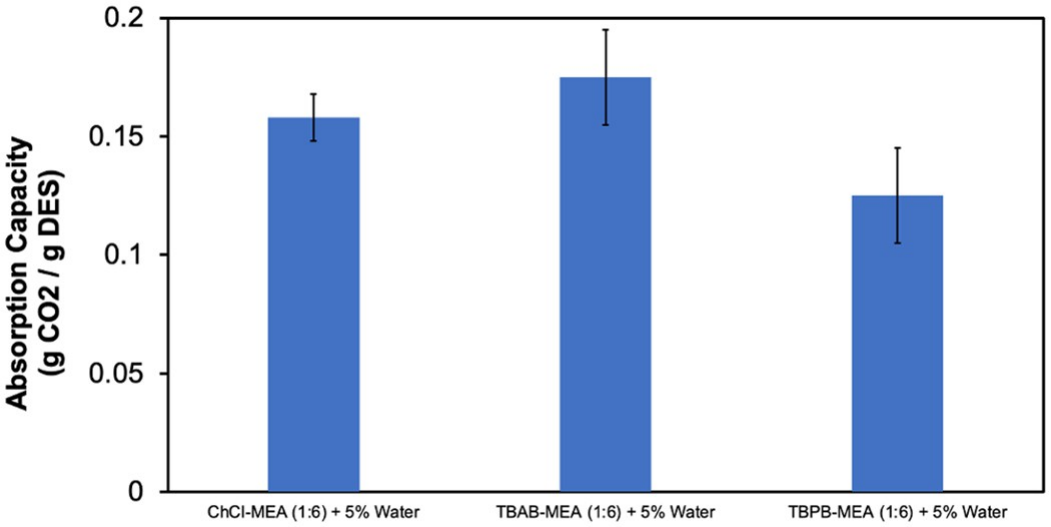
Effect of HBA type on CO_2_ absorption capacity of the MEA-based DESs + 5% water.

Capacity results using the microreaction calorimeter used for measuring CO_2_ absorption was first tested by measuring the absorption capacity of 30 wt% aqueous MEA and compared it with values reported in the literature. The obtained result, 0.12 g CO_2_ / g solvent, is in excellent agreement with that reported by Ali et al. [41], 0.1195 g CO_2_ / g solvent. Then, the CO_2_ absorption capacity of the prepared DESs was measured and compared with that of the corresponding binary DESs and 30 wt.% MEA aqueous solution. Each experiment has been repeated at least twice, the average value was considered and the standard deviation indicated by the error bars in each plot (see Figs 6–9 below).

#### Effect of the water content

Fig 6 illustrates the effect of water content in the ternary DESs on CO_2_ absorption capacity. Binary ChCl- MEA (1:6) DES showed a CO_2_ absorption capacity of 0.17 g/g.

The absorption capacity of the other two DESs decreased slightly: 0.158 g/g for the ChCl- MEA (1–6)—water (5%) and 0.155 g/g for the ChCl- MEA (1–6)—water (10%). The slight decrease in CO_2_ absorption with the increase of water concentration could be due to the decrease in the weight fraction of MEA, which has a high affinity for CO_2_. However, the decrease is comparatively small compared to the decrease in the viscosity and consequently there is a faster mass transfer of CO_2_ in the ternary DESs. Compared to the significant decrease in viscosity due to the inclusion of water in the DES, this slight decrease in absorption capacity can be considered insignificant. Consequently, these novel amine-based ternary DESs show great potential for industrial use due to their high CO_2_ absorption capacity in addition to their relatively low viscosity.

The effect of water concentration on ChCl- DEA (1:6)-water (5%) was similar to that of ChCl- MEA. However, the decrease of absorption capacity with the increase of water was relatively significant. It can also be seen from Fig 6 that the CO_2_ absorption capacity of the DESs based on MEA and DEA was higher than that of the 30 wt% aqueous MEA. This is because the CO_2_ absorption by the 30 wt% aqueous MEA is achieved by chemical absorption only, while the CO_2_ absorption by the DESs is achieved by chemical and physical absorption [42].

On the other hand, the results for ChCl-MDEA (1:6) DES and ChCl-MDEA (1–6)—water (5%) showed an opposite effect to that of the two aforementioned DESs. In this case, ChCl-MDEA (1:6) DES had an absorption capacity of 0.01 g/g, while ChCl-MDEA (1–6)-water (5%) had an absorption capacity of 0.024 g/g. This increase in CO_2_ absorption capacity is due to the fact that the absorption of CO_2_ by tertiary amines in the absence of water occurs only by a physical mechanism. However, when water is present, the reaction CO_2_ with the tertiary amine to form carbamate becomes possible, and accordingly the CO_2_ absorption capacity increases due to the additional chemical absorption [43].

#### Effect of the HBA:HBD molar ratio

The effect of HBA:HBD molar ratio on CO_2_ absorption capacity is shown in Fig 7. It can be observed that the absorption capacity increases from 0.158 g/g to 0.162 g/g and 0.17 g/g, respectively, when the molar ratio for ChCl- MEA- water (5%) decreased from 1:6 to 1:8 to 1:10. This trend is expected since the availability of MEA for reaction with CO_2_ increases with the decrease of the molar ratio in DES, as mentioned earlier. In addition, the viscosity, and thus the mass transfer rate, decreases as the ratio of MEA in DES increases.

#### Effect of the type of HBA and HBD

The CO_2_ absorption capacity when changing the HBD, namely MEA, DEA and MDEA with ChCl as HBA, and fixing the molar ratio to 1:6 and 5 wt.% water is shown in Fig 8.

It can be seen that ChCl- MEA (1:6)-water (5%) has the highest absorption capacity, while ChCl-MDEA (1:6)-water (5%) has the lowest absorption capacity. This can be explained by the fact that MEA has the highest CO_2_ solubility among the 3 amines due to its lower viscosity, which improves mass transfer in the absorption process. On the other hand, as mentioned earlier, MDEA requires water for the chemical absorption of CO_2_. However, as water was added during the preparation of DES, it formed complex hydrogen bonds with the components of DES. Therefore, not all of the water is available for reaction with CO_2_, so the CO_2_ absorption capacity is not as high as that of MEA and DEA based DESs.

In addition, the effect of HBA on the CO_2_ absorption capacity was investigated. Three different HBAs, namely ChCl, TBAB, and TBPB, were used with MEA as HBD, a molar ratio of 1:6, and 5 wt% water. It can be seen from Fig 9 that TBAB-MEA (1:6)- water (5%) had the highest absorption capacity and TBPB-MEA (1:6)- water (5%) had the lowest absorption capacity. According to the results reported in the literature, there is no clear trend in the difference of CO_2_ absorption between ammonium-based and phosphonium based DESs [41, 44–48]. The solubility depends on many factors including the strength of hydrogen bonding within the DES structure, molar ratio, molar volume, free volume, molecular weight and alkyl chain length [44]. More work is needed to reach a solid conclusion.

#### Prediction of CO_2_ solubility using COSMO-RS

COSMO-RS was used to determine the effects of temperature and pressure changes on the CO_2_ absorption of DESs. In addition, the effects of the molar ratio of HBA:HBD and the nature of HBD and HBA were investigated and compared with the corresponding experimental results. CO_2_ solubility was predicted and compared with experimental values to determine the ability of COSMO-RS is in quantitatively predicting CO_2_ solubility. To proceed with the COSMO-RS simulations, the components involved were first created in the TURBOMOLE program using TmoleX software. Then, COSMOthermX was used to predict the CO_2_ solubility of the DESs [33].

**Fig 10:**
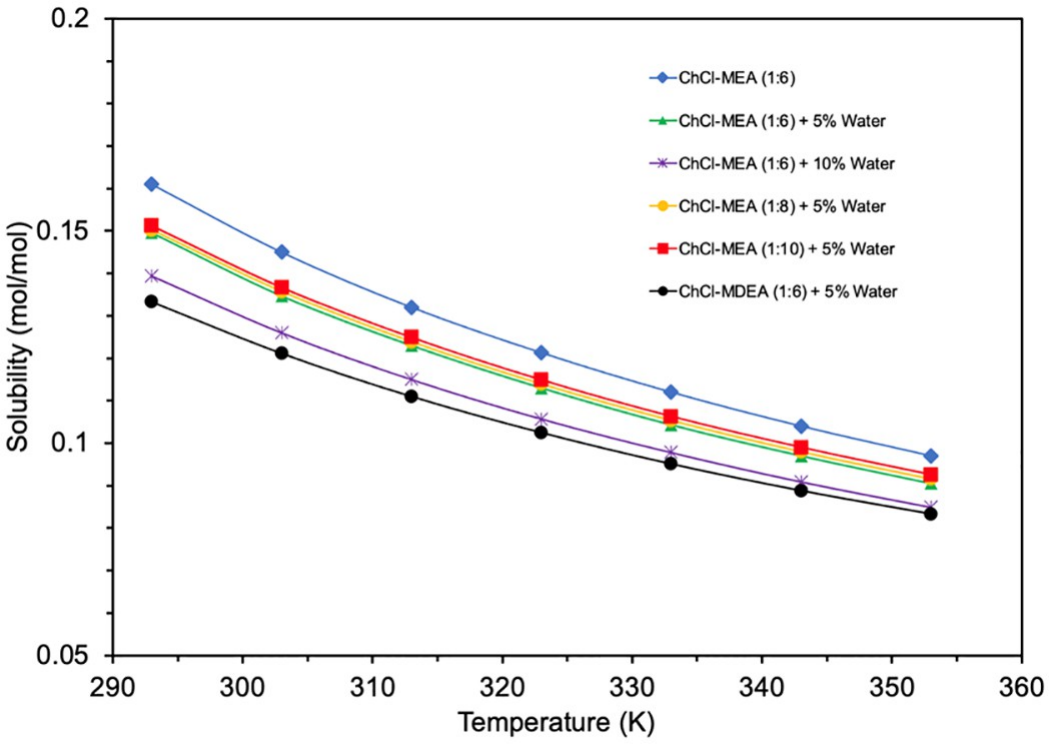
Effect of temperature on CO_2_ solubility of amine-based DESs predicted by COSMO-RS.

The effect of temperature change in the range between 293 K and 353 K on CO_2_ solubility is shown in Fig 10. It is clear that the CO_2_ solubility of all DESs decreases with increasing temperature.

**Fig 11:**
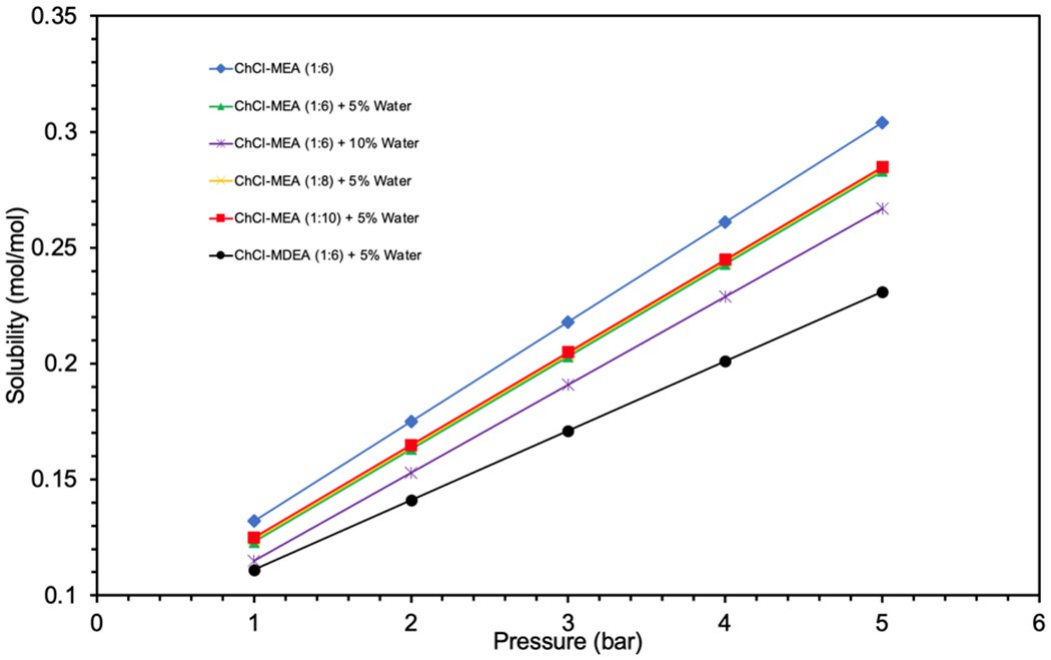
COSMO-RS prediction for pressure effect on CO_2_ solubility of amine-based DESs.

This trend is due to the fact that as the temperature increases, the molecules gain more kinetic energy and can move more freely, which makes the CO_2_ molecules more likely to escape into the gas phase. Moreover, ChCl- MEA (1:10)-water (5%) showed the highest CO_2_ solubility among the tested DESs with ChCl as HBA, while ChCl-MDEA (1:6)- water (5%) showed the lowest CO_2_ solubility. Moreover, the solubility increases when the molar ratio of MEA:ChCl increases from 1:6 to 1:10. When comparing ChCl- MEA (1:6)- water (5%), ChCl- MEA (1:6)- water (10%) and ChCl- MEA (1:6), it is also found that the solubility of CO_2_ decreases with increasing water content. All these results are in agreement with the experimental results and reflect the accuracy of this software in qualitatively predicting such factors [49]. Moreover, the effect of pressure changes in the range between 1 bar and 5 bar on CO_2_ solubility is illustrated in Fig 11. As expected, the CO_2_ solubility increases with increasing pressure. This relationship is consistent with Henry’s law [50].

**Fig 12:**
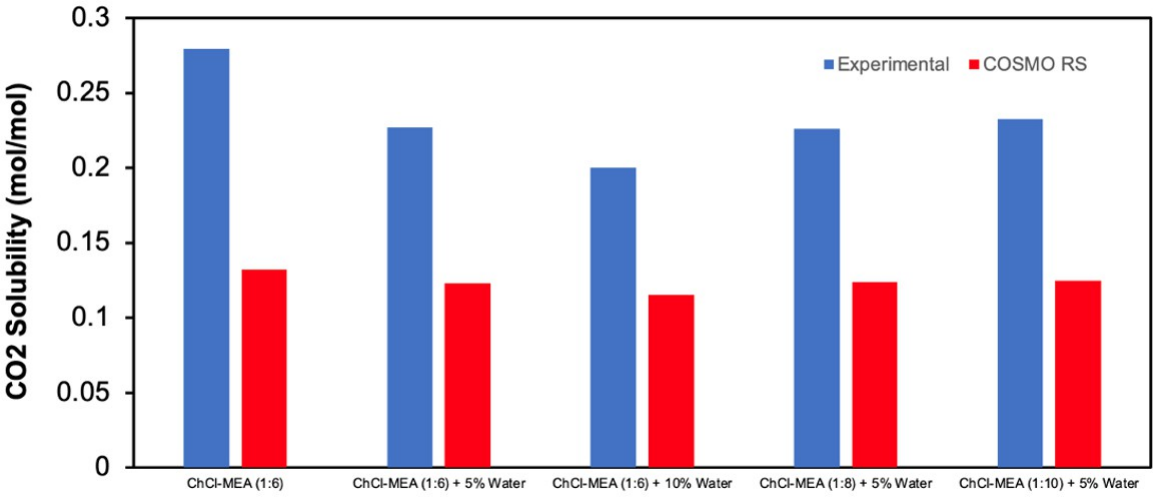
Experimental vs COSMO-RS values for the CO_2_ solubility of the studied DESs.

Fig 12 shows a comparison of the CO_2_ solubility values predicted by COSMO-RS with the corresponding experimental values at 40°C for selected DESs.

From Fig 12, it can be seen that the experimental values were higher than those predicted by COSMO-RS for all DESs tested. However, both the experimental and predicted data show the same trend in terms of changes in water content and molar ratio. The relative deviation, (*x*^*exp*^ − *x*^*COSMO RS*^)/*x*^*exp*^) × 100%, between experimental and predicted values is significant. For example, in the case of ChCl- MEA (1:6), the relative deviation is about 52.7%. This indicates that COSMO-RS can be used qualitatively but not quantitatively to predict CO_2_ solubility. Most likely, this is due to the fact that COSMO-RS takes into account the physical absorption of CO_2_ only, but not the chemisorption of CO_2_ in the amines.

### Modeling the DESs viscosity using the Vogel-Fulcher-Tammann (VFT) model

There are several models that describe the effect of temperature on the viscosity of liquids. In this study, the temperature-dependent viscosity data for the different selected DESs were fitted according to the Vogel-Fulcher-Tammann-Hesse (VFTH) equation (Eq 1) [51–53]:

$$\eta =A\cdot \;exp\;\left[\;\frac{B}{T-T_{0}}\right]$$ (1)

This three-parameter model has been shown to be the most accurate equation for estimating the viscosity of fluids, especially in the low temperature range [54].

The fitting results were obtained using MATLAB Fitting Toolbox. The fitting parameters *A*, *B*, and *T*_*0*_, as well as the regression coefficient *R*^*2*^ and the average absolute relative deviation (AARD) are shown in Table 4. With a correlation coefficient *R*^*2*^ that is close to 1, an excellent fit is achieved. Moreover, the AARD does not exceed 3.2%, indicating excellent agreement between the experimental data and the VFTH equation.

**Table 4:** Parameters of the VFTH equation for temperature dependence of viscosity (mPa.s), together with the correlation coefficients and relative deviation.

DES type	A (mPa.s)	B (K)	T_0_ (K)	R^2^	AARD (%)
ChCl-MEA (1:6)	0.03494	948.1	164.4	1	0.32
ChCl-MEA (1:6) + 2.5% Water	0.02718	1012	158.8	1	0.45
ChCl-MEA (1:6) + 5% Water	0.0181	1138	148.9	1	0.71
ChCl-MEA (1:6) + 7.5% Water	0.01589	1206	140.9	0.9998	2.00
ChCl-MEA (1:6) + 10% Water	0.005001	1518	124.6	0.9999	2.17
ChCl-MEA (1:6) + 12.5% Water	0.07434	657.3	187.8	0.9998	1.07
ChCl-MEA (1:8) + 5% Water	0.04552	852.6	169	0.9997	2.47
ChCl-MEA (1:10) + 5% Water	0.06876	720.2	178.9	0.9998	2.21
ChCl-DEA (1:6) + 5% Water	0.005101	1699	143.4	1	0.87
ChCl-MDEA (1:6) + 5% Water	0.01278	1380	142.6	1	0.54
TBAB-MEA (1:6) + 5% Water	0.04316	792.2	187.1	0.9991	2.04
TBPB-MEA (1:6) + 5% Water	0.0837	691.8	184.8	0.9996	3.22

### Thermal stability of DESs

Thermal degradation of alkanolamines in general and MEA in particular leads to significant solvent loss in the stripping section of CCS plants during the regeneration step, where the temperature is usually around 120°C, contributing to an increase in operating costs. At such temperature, amines tend to react irreversibly with acid gasses such as CO_2_, H_2_S, SO_x_, and NO_x_, leading to solvent decomposition [55, 56]. Thermal degradation of amines also leads to the formation of undesirable pollutants such as nitrosamines and nitramines, which cause serious environmental and health problems [57, 58]. Moreover, the degradation of amines can lead to other problems such as corrosion, foaming, and fouling of the stripping columns, in addition to the decrease in the absorption capacity of the solvents [59–61].

**Fig 13:**
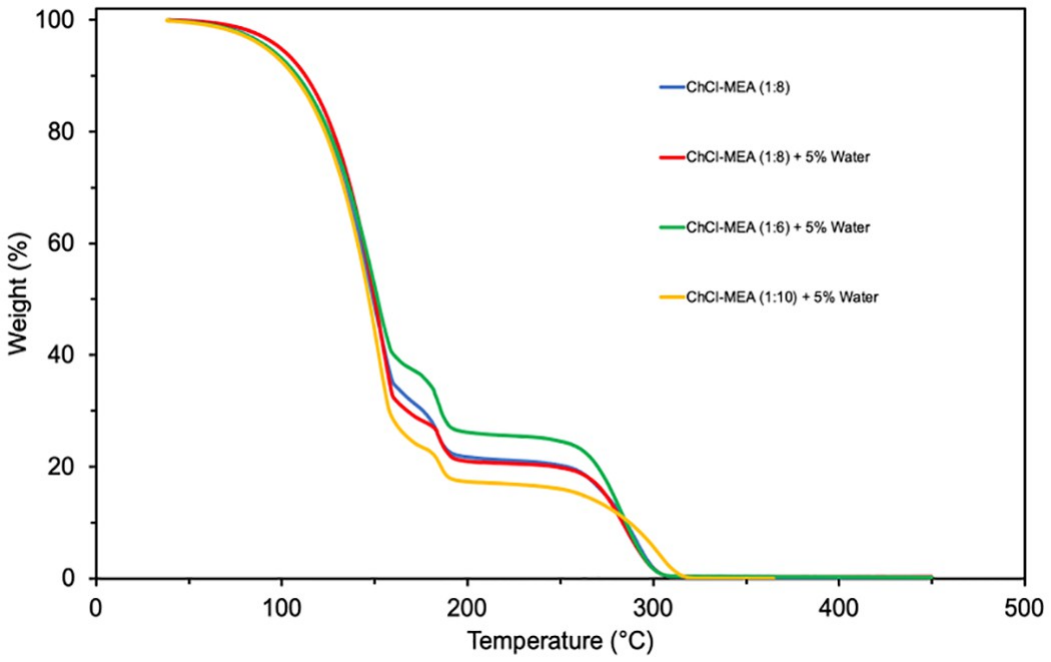
Effect of water content (5%) on the thermal stability of ChCl-MEA DESs.

In this study, the thermal stability of selected DESs was measured using the Perkin Elmer Thermogravimetric Analyzer (TGA) 4000 by plotting the weight fraction of DES against temperature, as shown in Fig 13.

The main objective is to evaluate the effect of water content on the thermal stability of amine-based ternary DESs. It was found that the degradation temperature of ChCl- MEA (1:8) is about 125°C, which is much higher than that of MEA with a degradation temperature of about 65°C as reported by Adeyemi et al. [36]. This confirms the clear advantage that amine-based DESs have over stand-alone amines in terms of thermal stability. Moreover, the degradation temperature of the water-containing DESs, namely ChCl- MEA (1:6)-water (5%), ChCl- MEA (1:8)- water (5%), and ChCl- MEA (1:10)- water (5%), was similar. These results indicate that the water did not evaporate at its normal boiling point. This is because the water, when added during the preparation of DES, becomes part of the structure of DES by forming hydrogen bonds with the other constituents of the DES. Such behavior is extremely advantageous for post-combustion process, where the regeneration temperature is around 120°C [62]. Moreover, the degradation temperature of all DESs is higher than that of 30 wt% aqueous MEA, which has a thermal degradation temperature of about 105°C according to Kang et al. [63]. This is due to the complex and strong hydrogen bonds between the constituents of DESs, in addition to their negligible vapor pressure, which reduces the solvent loss due to degradation [64].

## Conclusion

Novel amine-based ternary DESs having water as a constituent were prepared for the potential use for CO_2_ absorption. The physicochemical properties such as melting point, viscosity and density of the DESs were measured. It was found that the viscosity of the ternary DESs decreased up to 25% which is highly favorable for CO_2_ absorption. The thermal stability of these ternary DESs, determined by TGA analysis, was found to be similar to that of binary DESs, with an initial degradation temperature of about 125°C compared to 105°C of 30 wt. % MEA aqueous solution. This indicates that the water in these DESs is not free and that it is combined with the HBA and HBD with strong hydrogen bonding. The measured CO_2_ absorption capacity for the ternary DESs were found to be between 0.155 and 0.17 (g CO_2_ / g DES) for MEA based DESs, which is higher than that of 30 wt. % aqueous MEA (0.12 g CO_2_ / g solvent). CO_2_ solubility in the DESs was predicted using COSMO-RS and compared with the experimental values. It was found that COSMO-RS is a suitable tool for qualitative prediction of CO_2_ solubility in DESs, since it correctly represents the trend resulting from the effects of changing different factors on CO_2_ solubility compared to experimental results.

In addition, the influence of water content, molar ratio of HBA to HBD, and type of HBD and HBA on the physiochemical properties and CO_2_ absorption of the ternary DESs were investigated. The results showed that ChCl- MEA (1:6), (1:8) and (1:10)- water (5 and 10%) were the best candidates for CO_2_ capture in terms of their CO_2_ absorption capacity and thermal stability.

In summary, the use of water as constituent in the amine based ternary DESs reduced the viscosity and did not affect the CO_2_ absorption capacity or the thermal stability compared to binary amine based DESs. This makes the novel ternary DESs a potential solvent for CO_2_ capture. However, more work is needed to measure the heat needed for regeneration.

## Supporting information

S1 Table Viscosities of the prepared DESs at different temperatures. (DOCX)

S2 Table Densities of the prepared DESs at different temperatures. (DOCX)

S1 Appendix Experimental protocol. (DOCX)
