# Supplementary material for: Experimental investigation of novel ternary amine-based deep eutectic solvents for CO2 capture
Source: PLoS One. 2023 Jun 23;18(6):e0286960. doi: 10.1371/journal.pone.0286960 (PMC10289352; doi:10.1371/journal.pone.0286960)
Supplement: S1 Table — (DOCX) [file pone.0286960.s001.docx]

**S1 Table. Viscosities of the prepared DESs at different temperatures.**

|  |  |  | | **Viscosity (mPa.s)** | |  | |  | |
| --- | --- | --- | --- | --- | --- | --- | --- | --- | --- |
| **DES** | **293 K** | | **298 K** | **313 K** | **333 K** | | **353 K** | |  |
| ChCl-MEA (1:6) | 55.62 | | 42.31 | 20.57 | 9.66 | | 5.38 | |  |
| ChCl-MEA (1:6) + 2.5 % Water | 51.00 | | 38.99 | 19.16 | 9.02 | | 5.02 | |  |
| ChCl-MEA (1:6) + 5 % Water | 48.80 | | 37.40 | 18.70 | 8.62 | | 4.83 | |  |
| ChCl-MEA (1:6) + 7.5 % Water | 43.91 | | 34.42 | 17.55 | 8.06 | | 4.50 | |  |
| ChCl-MEA (1:6) + 10 % Water | 41.23 | | 31.99 | 15.83 | 7.07 | | 4.11 | |  |
| ChCl-MEA (1:6) + 12.5 % Water | 38.21 | | 29.10 | 13.88 | 6.98 | | 4.00 | |  |
| ChCl-MEA (1:8) + 5 % Water | 44.05 | | 33.92 | 17.12 | 7.78 | | 4.46 | |  |
| ChCl-MEA (1:10) + 5 % Water | 37.80 | | 29.22 | 14.70 | 7.19 | | 4.01 | |  |
| ChCl-DEA (1:6) + 5 % Water | 434.14 | | 301.20 | 113.54 | 39.53 | | 17.28 | |  |
| ChCl-MDEA (1:6) + 5 % Water | 123.16 | | 91.77 | 41.96 | 17.81 | | 9.14 | |  |
| TBAB-MEA (1:6) + 5 % Water | 75.89 | | 55.82 | 22.09 | 9.75 | | 5.15 | |  |
| TBPB-MEA (1:6) + 5 % Water | 50.05 | | 38.23 | 18.56 | 8.55 | | 4.65 | |  |
