# Supplementary material for: Experimental investigation of novel ternary amine-based deep eutectic solvents for CO2 capture
Source: PLoS One. 2023 Jun 23;18(6):e0286960. doi: 10.1371/journal.pone.0286960 (PMC10289352; doi:10.1371/journal.pone.0286960)
Supplement: S2 Table — (DOCX) [file pone.0286960.s002.docx]

**S2 Table. Densities of the prepared DESs at different temperatures.**

|  |  |  | | **Density (g/cm^3^)** | |  | |  | |
| --- | --- | --- | --- | --- | --- | --- | --- | --- | --- |
| **DES** | **293 K** | | **298 K** | **313 K** | **333 K** | | **353 K** | |  |
| ChCl-MEA (1:6) | 1.0511 | | 1.0475 | 1.0367 | 1.0225 | | 1.0084 | |  |
| ChCl-MEA (1:6) + 2.5 % Water | 1.0522 | | 1.0485 | 1.0379 | 1.0237 | | 1.0096 | |  |
| ChCl-MEA (1:6) + 5 % Water | 1.0530 | | 1.0498 | 1.0392 | 1.0251 | | 1.0109 | |  |
| ChCl-MEA (1:6) + 7.5 % Water | 1.0535 | | 1.0498 | 1.0384 | 1.0253 | | 1.0110 | |  |
| ChCl-MEA (1:6) + 10 % Water | 1.0540 | | 1.0504 | 1.0400 | 1.0260 | | 1.0118 | |  |
| ChCl-MEA (1:6) + 12.5 % Water | 1.0540 | | 1.0503 | 1.0400 | 1.0260 | | 1.0118 | |  |
| ChCl-MEA (1:8) + 5 % Water | 1.0434 | | 1.0396 | 1.0287 | 1.0141 | | 0.9995 | |  |
| ChCl-MEA (1:10) + 5 % Water | 1.0425 | | 1.0388 | 1.0278 | 1.0131 | | 0.9984 | |  |
| ChCl-DEA (1:6) + 5 % Water | 1.1011 | | 1.0979 | 1.0891 | 1.0768 | | 1.0644 | |  |
| ChCl-MDEA (1:6) + 5 % Water | 1.0590 | | 1.0553 | 1.0450 | 1.0311 | | 1.0170 | |  |
| TBAB-MEA (1:6) + 5 % Water | 1.0372 | | 1.0334 | 1.0236 | 1.0087 | | 0.9935 | |  |
| TBPB-MEA (1:6) + 5 % Water | 1.0403 | | 1.0364 | 1.0253 | 1.0103 | | 0.9951 | |  |
