# Supplementary material for: Experimental investigation of novel ternary amine-based deep eutectic solvents for CO2 capture
Source: PLoS One. 2023 Jun 23;18(6):e0286960. doi: 10.1371/journal.pone.0286960 (PMC10289352; doi:10.1371/journal.pone.0286960)
Supplement: S1 Appendix — (DOCX) [file pone.0286960.s003.docx]

# Appendix: Experimental protocol

During the start of the process, the power signal fluctuates for a few minutes until it reaches a stable value. Once the power signal reaches a steady state, the CO_2_ cylinder is opened and the flow rate is set to 0.5 ml/min. During the CO_2_ absorption phase, the power signal increases to a maximum value and then starts to decrease, resulting in a spike in the graph due to the exothermic process. After a few hours, the power signal stabilizes as CO_2_ absorption is complete. The CO_2_ cylinder is then closed and the sample is removed from the calorimeter and weighed. The mass of the sample is then measured and subtracted from its mass prior to the experiment to determine the mass of absorbed CO_2_. The mass of absorbed CO_2_ is then divided by the mass of the initial sample to determine the CO_2_ absorption capacity in grams of CO_2_ per gram DES. The CO_2_ heat of absorption of the DESs is measured with the same equipment, using an empty reference vial to reduce the heat difference between the reference and the sample. The heat of absorption is determined by the μRC control and analysis software after the run is completed. It is evaluated by integrating the area under the Power vs. Time graph, which gives the value in joules. This value is converted to kJ/mol, taking into account the mass of CO_2_ absorbed.
